# Knowledge of local snakes, first‐aid and prevention of snakebites among community health workers and community members in rural Malawi: A cross‐sectional study

**DOI:** 10.1111/tmi.14071

**Published:** 2024-12-17

**Authors:** Moses Banda Aron, Fabien Munyaneza, Anat Rosenthal, Luckson Dullie, Ralf Krumkamp, Enoch Ndarama, Bright Mailosi, Jürgen May, Basimenye Nhlema, Clara Sambani, Deborah Hosemann, Jade Rae, Paul Rahden, Jörg Blessmann, Benno Kreuels

**Affiliations:** ^1^ Neglected Diseases and Envenoming Bernhard Nocht Institute for Tropical Medicine Hamburg Germany; ^2^ Partners In Health/Abwenzi Pa Za Umoyo Neno Malawi; ^3^ Department of Health Policy and Management Ben‐Gurion University of the Negev Beersheba Israel; ^4^ Department of Infectious Disease Epidemiology Bernhard Nocht Institute for Tropical Medicine Hamburg Germany; ^5^ German Center for Infection Research, Hamburg, Borstel Lübeck Germany; ^6^ Neno District Health Office Neno Malawi; ^7^ Department of Global Health and Social Medicine Harvard Medical School Boston Massachusetts USA; ^8^ Tropical Medicine II University Medical Center Hamburg‐Eppendorf (UKE) Hamburg Germany; ^9^ Department of Research Ministry of Health Lilongwe Malawi; ^10^ Division for Tropical Medicine, I. Department of Medicine University Medical Center Hamburg Hamburg Germany; ^11^ Department of Medicine, School of Medicine Kamuzu University of Health Sciences Blantyre Malawi

**Keywords:** community members, first‐aid, knowledge, local snakes, prevention, snakebite

## Abstract

**Objective:**

Snakebite envenoming remains a public health threat in many tropical countries. While community knowledge of local snakes and snakebite first‐aid and prevention are needed to reduce snakebite incidence and improve the outcomes for snakebite patients, it is poor in many communities. We assessed community health workers and community members regarding their knowledge on local snakes, snakebite first‐aid and prevention in Neno district, Malawi.

**Methods:**

In November 2022, we conducted a cross‐sectional survey among 312 community health workers and 379 community members in the Neno District of Malawi to assess their knowledge of snake identification, snakebite first‐aid, and prevention. Different questions were asked in these sections and summarised as linear scores ranging from 0% to 100%. Scores of 0%–49%, 50%–70%, and >70% were considered inadequate, fairly adequate, and adequate, respectively. Along with data collected during knowledge assessments, the socio‐demographic characteristics of participants were collected. To assess knowledge differences between community health workers and community members, Pearson's chi‐square or Fisher's exact tests were used, and linear regression was calculated to investigate possible predictors of knowledge.

**Results:**

Overall, 66.6% of participants were females with a median age of 39 (IQR = 30–48) years. Of the 89% (*n* = 615) who agreed to view snake pictures, only 1.3% had adequate snake identification knowledge. Less than 5% (*n* = 33) had adequate knowledge of first aid measures, and 14.3% (*n* = 99) had adequate knowledge of prevention practices. Overall, less than 1% (*n* = 3) had adequate knowledge across the three assessment sections, with no significant difference between community health workers (*n* = 1, 0.3%) and community members (*n* = 2, 0.5%) (*p* > 0.949).

**Conclusion:**

Both community health workers and community members had inadequate knowledge regarding local snake species, first aid for snakebites and prevention measures. The effect of awareness campaigns and other education initiatives could be explored to help improve these gaps.

## INTRODUCTION

Snakebite envenoming is one of the 21 neglected tropical diseases (NTD) and a public health threat in many tropical and subtropical countries [1, 2]. The World Health Organization (WHO) estimates that over 5 million snakebite cases occur annually, with 1.8–2.7 million resulting in envenoming [2] and many victims suffering from long‐term complications [3, 4, 5]. Approximately 314,000 snakebite envenoming cases occur in the Sub‐Saharan Africa region, resulting in between 5900 and 14,600 amputations and 7000 and 32,000 deaths [6]. However, studies have suggested that, due to a lack of reliable data in the region, these estimates likely underestimate the problem [7, 8, 9]. Reducing the morbidity and mortality from snakebites relies on effective prevention measures, first aid practices, and proper treatment. Nonetheless, these approaches require adequate knowledge of the topic, which is poor in many settings [10, 11, 12].

Knowledge of snakes responsible for envenoming is important to manage and treat snakebites effectively. However, a systematic review of 150 studies across 35 countries among healthcare workers and the general population showed ongoing challenges in accurately identifying snakes and determining whether they were venomous [13]. More than 70% of snakebite victims report seeing the culprit snake or killing it and bringing it to the health facility. Nevertheless, misidentification among community members and healthcare workers remains common [13, 14].

Appropriate first‐aid methods are crucial to prevent death and disability from snakebites [15, 16, 17]. In many communities, information on providing adequate first‐aid to prevent complications and poor outcomes of envenoming is lacking [10]. World Health Organisation recommends that in case of snakebite victims should be reassured, constricting clothes should be removed and the affected limb should be immobilised if possible until a health facility is reached. However, unsafe practices, including using tourniquets, suction, application of various herbal blends or black stones, and incisions as first aid for snakebites, are still widely used [15, 18, 19, 20]. While black stones have been widely used traditionally in the management of snakebites, there is still no evidence of their effect [21].

Successful snakebite prevention relies on the knowledge and use of recommended prevention practices. Preventive measures like clearing high grass and debris around houses, using torches and proper footwear, particularly at night outside along with using raised beds and mosquito nets can help prevent snakebites. Animals should be kept outside the living areas [22]. However, several studies have suggested that, while community members are aware of recommended snakebite first aid and prevention methods, they often practice unsafe first aid and unproven preventive strategies [10, 23, 24]. Community engagement through awareness campaigns and advocacy messages is needed to improve knowledge and practices [10, 25, 26, 27].

Community health workers (CHWs), recruited from the community to provide healthcare services without formal medical training [28], are used to improve treatment‐seeking behaviour by delivering preventive education and acting as a link to primary health facilities through case referrals and patient follow‐ups [29]. Studies have shown the valuable role of CHWs as ‘frontline warriors’ within communities against various diseases, including NTDs [30, 31]. Nevertheless, there remains a lack of evidence on their role in the fight against snakebites in many countries including Malawi [32, 33], with little data on CHWs' knowledge regarding snake identification, first aid and prevention [12, 34]. In two previous studies, we evaluated healthcare workers' and traditional healers' snakebite knowledge, experiences, and management practices in Malawi [35, 36]. At the health facility level, knowledge of the symptoms associated with envenomation allows for targeted use of antivenom, and a syndromic approach can be helpful when polyvalent antivenom is available [37]. In a community setting in Malawi, where antivenom is rarely available and access to health care is limited and costly, well‐trained CHWs and other community members with good knowledge of local snakes, clinical danger signs and first aid can improve snakebite prevention, provide first aid and advise on the need for referral, while preventing unnecessary presentations to health facilities or traditional healers [32].

The study aimed to assess the knowledge of CHWs regarding snake identification, first aid techniques, and preventive measures and compare this knowledge to that of other community members to guide the delivery of community awareness campaigns and improved education among CHWs.

## METHODS

### Study design and setting

This cross‐sectional study was conducted in November 2022 in Neno District in Malawi. In reporting, we followed the Strengthening the Reporting of Observational Studies in Epidemiology (STROBE) guidelines (Data S1, STROBE checklist).

Malawi is a low‐income country in southern Africa with an estimated population of 18.4 million people as of 2022 [38]. Neno District is located southwest of Malawi and is one of the country's most remote and difficult‐to‐access districts. In 2022, the estimated mid‐year population in the district was 150,211 people [38]. For over 94% of households, the main source of income comes from smallholder subsistence farming [39, 40]. Only 8% of households have access to electricity, and nearly everyone depends on wood or charcoal as a source of energy for cooking [41]. There are 15 health facilities in Neno District, two secondary‐level hospitals and 13 primary‐level healthcare centres.

### Study population

Since 2007, Partners in Health (PIH), known locally in Malawi as Abwenzi Pa Za Umoyo (APZU), has supported the Ministry of Health in the operation of health facilities and in the training and deployment of CHWs to provide services to rural communities in Neno District [42]. CHWs have been vital for community‐based primary care delivery, focusing on prevention, patient support, and linkage to health facilities [39, 40]. CHWs are recruited from their villages and undergo a comprehensive 5‐day training. Each CHW oversees 20 to 40 households within an assigned village [39, 40]. The responsibilities of CHWs include community education and health screening, primarily focused on eight areas characterised by high disease burden or medical relevance. These include HIV, tuberculosis (TB), non‐communicable diseases (NCDs), sexually transmitted infections (STIs), child health, malnutrition, family planning and maternal and neonatal health [39]. For this study, we targeted all 1233 CHWs and community members above 18 years of age living in the 169 registered villages in Neno District.

### Sample size and sampling techniques

We obtained a list of CHWs from the PIH/APZU Community Health Department, indicating 1233 CHWs. For this study, to obtain a reasonable representation of this population with the available resources, we aimed to survey approximately a quarter of the CHWs (*n* = 312) in the district with representation across all 169 villages. To achieve this, we randomly selected one CHW per village using a random number generator. In a second step, to select the remaining 143 CHWs required to achieve the targeted sample size of 312, we randomly sampled 143 further CHWs with probability proportional to the number of CHWs per village.

Community members were selected from the households attended by the recruited CHW. Per CHWs who served 40 households or less, we recruited one household using a random walk from the location of the CHWs household. The walk's direction (north, east, south, west) and duration (2 or 5 min) were randomly selected for each household. For the 67 CHWs who served more than 40 households, we included two households instead of one from the surrounding area, resulting in a total of 379 community members who participated in the study. Per selected household, we interviewed one person who volunteered to participate in the survey.

### Data collection

We developed a questionnaire after reviewing the available literature on healthcare workers and community members' knowledge of snake identification, first aid, and prevention measures (Data S2) [11, 24, 35, 43]. The questionnaire was designed to collect information data on socio‐demographic characteristics and information on knowledge of (i) local snake species; (ii) first aid after snakebites as recommended by the WHO including moving the victim to safety from the area where they might be bitten again, removing constricting clothing, rings, bracelets, bands, shoes and so forth from the bitten limb, immobilise the whole patient, especially the bitten limb using a splint or sling, avoid the many harmful and time‐wasting application of traditional treatments among others [44]; (iii) the WHO‐recommended practices for snakebite prevention include cleaning and clearing bushes and debris lying on the ground, using a torch outside at night, using protective equipment, for example, safety boots, sleeping off the ground and using mosquito nets during sleep, among others [22, 24]. To assess snake identification skills participants were shown six photos of snakes and were asked to name the snake and classify the snake as venomous or non‐venomous (Data S3). Participants with snake phobia could opt not to view these pictures. Venomous snakes included the Puff adder (*Bitis arietans*), Black mamba (*Dendroaspis polylepis*), Oates' vine twig (*Thelotornis oastesi*), and Mozambique spitting cobra (*Naja mossambica*). Non‐venomous snakes included the common house snake (*Boaedon capensis*) and the spotted bush snake (*Philothamnus semivariegatus*). All snake species in the photos are common in Malawi except the Black mamba [45, 46]. The questionnaire was translated into ‘Chichewa’, Malawi's official local language [47]. We recruited and trained six research assistants from the district who knew the community well. The questionnaire was piloted in 15 villages, and minor adjustments were made. Data were collected using Dimagi's CommCare platform [47].

### Data analysis

Our analysis was based on previously published studies done in Cameroon, Ghana and Bhutan [24, 48, 49] where a correct response to the question was assigned one point and choosing a wrong or an ‘I don't know’ option attracted no point. In our context, for example in the snake identification section, if picture ‘A’ is a puff alder and the respondent mentions it correctly, was given 1 point. If they mentioned a wrong name or ‘don't know’, no point was given. Similarly, WHO does not recommend ‘Tie the affected area to stop blood circulation (tourniquet)’ following snakebite, if the participant responded ‘no’, 1 point was given otherwise they responded with a ‘yes’ or ‘don't know’, no point was given. The percentage of correct responses was calculated separately for each questionnaire section (i.e., snake identification, first aid, and snakebite prevention) due to the difference in number of questions. In addition, an overall knowledge score was calculated by combining the percentage scores across the three sections. The resulting percentage was then classified as ‘inadequate knowledge’ (0%–49% correct), ‘fairly adequate knowledge’ (50%–70% correct) and ‘adequate knowledge’ (>70% correct). Descriptive data analysis was performed by summarising categorical variables using counts and percentages, and continuous variables were summarised using the median and interquartile range (IQR). Mann–Whitney test was used to compare the median knowledge scores between community members and CHWs. Pearson's chi‐square test or Fisher's exact test (if observed counts were less than five within at least one category) were used to assess associations in responses between CHW and community members. Finally, we used bivariable and multivariable linear regressions to investigate associations between the overall knowledge score and covariates, including respondent type (community member or CHW) and respondent characteristics (sex, age, childhood residence and highest education level). Data cleaning and analysis were performed with R Project for Statistical Computing (version 4.2.2) [50].

### Ethical considerations

We obtained ethical clearance from the Malawi National Health Science Research Committee (protocol number: NHSRC/21/10/2816; dated 26 November 2021). Before administering the questionnaire, we obtained written informed consent from the Community Health Workers and community members. Participants were free to withdraw from the study at any point in time. Data were de‐identified before analysis, kept securely by the investigator and shared with the co‐authors in anonymised form in a password‐protected file.

## RESULTS

### Socio‐demographic characteristics of study participants

We interviewed 691 individuals, which included 379 community members and 312 CHWs. Four hundred and sixty (66.6%) were female with a median age was 39 years (IQR = 30–48), and 95% (*n* = 658) grew up in rural areas. Among community members, 77.3% (*n* = 293) had eight or fewer years of education, compared to 42.9% (*n* = 134) in the CHW group. Overall, 63.1% (*n* = 427) reported to travel more than an hour to reach the nearest health facility (Table 1).

**TABLE 1:** Socio‐demographic characteristics of individuals included in knowledge questionnaires in Neno District, Malawi.

	Type of participant; *n* (%)		
Variable	Community member	Community health worker	Overall
Number of respondents	379 (54.8)	312 (45.2)	691 (100)
Sex of respondents			
Female	260 (68.6)	200 (64.1)	460 (66.6)
Male	119 (31.4)	112 (35.9)	231 (33.4)
Age in years (Median (IQR))	37 (27–50)	41 (34–46)	39 (30–48)
Residence during childhood			
Rural	360 (95.0)	298 (95.5)	658 (95.2)
Urban	19 (5.0)	14 (4.5)	33 (4.8)
Highest education level			
No formal education/primary	293 (77.3)	134 (42.9)	427 (61.8)
Secondary school/tertiary	86 (22.7)	178 (57.1)	264 (38.2)
Marital status			
Single	24 (6.3)	4 (1.3)	28 (4.1)
Married living together	264 (69.7)	245 (78.5)	509 (73.7)
Divorced separated	45 (11.9)	37 (11.9)	82 (11.9)
Widowed	46 (12.1)	26 (8.3)	72 (10.4)
Time to a health facility			
Less than 30 min	56 (14.8)	56 (17.9)	112 (16.2)
Between 30 min and 1 h	49 (12.9)	71 (22.8)	120 (17.4)
More than 1 h	253 (66.8)	183 (58.7)	436 (63.1)
Don't know	21 (5.5)	2 (0.6)	23 (3.3)

### Community health worker training and health education in their communities

Of the 312 CHWs, 96.5% (*n* = 301) had not provided snakebite education to their communities, and four of the 11 CHWs who offered snakebite education did so only once in the year preceding the survey (Table 2).

**TABLE 2:** Snakebite training received by the surveyed community health workers.

	*n* (%)^a^
Years of work experience (Median (IQR))	7.0 (5.0–14.0)
Have you offered any education on snakebite in your community?	
Yes	11 (3.5)
No	301 (96.5)
How many education lessons have you offered in the community in the last year?^b^	
1	4 (36.4)
2	4 (36.4)
3	1 (9.1)
>3	2 (18.2)
Reporting health facility^c^	
Health centre	253 (81.1)
Hospital	59 (18.9)
Have you ever received any snakebite training^d^	
Yes	9 (2.9)
No	297 (95.2)
Can't remember	6 (1.9)
Time since the training	
≥6 months but less than 2 years	3 (33.3)
More than 2 years	6 (66.7)

Abbreviations: CHW, community health worker; IQR, interquartile range.

^a^ All 312 CHWs responded to questions on training.

^b^ Only asked if they offered training the previous year (positive answer to the previous question).

^c^ The health facility that they report or refer their clients to.

^d^ Formal training through workshop or professional.

CHWs had worked for a median of 7 years in their position (IQR = 5–14), and 95.2% of CHWs (*n* = 297) had never received any snakebite‐related training. Of the nine CHWs who reported training, six (66.7%) received training more than 2 years before the survey (Table 2). Four out of five CHWs (*n* = 253, 81.1%) were affiliated with one of the 12 health centres, and the remaining 59 were affiliated with Neno District Hospital and Lisungwi Community Hospital.

### Snake identification, first aid and snakebite prevention knowledge

Of the 89% (*n* = 615) respondents who agreed to view snake pictures, 57.4% had inadequate knowledge of naming and identifying venomous snakes from photos. Of the snake photos presented during the questionnaire, the Puff adder (96%, *n* = 592) and the Mozambique spitting cobra (82%, *n* = 506) were most often correctly identified as venomous. However, 66% of respondents (*n* = 408) misidentified the common house snake and spotted bush snake (80%, *n* = 495) as venomous, and 70% (*n* = 474) of all respondents considered all snakes venomous (Data S4).

Three hundred and fifty‐three individuals had fairly adequate knowledge of first aid practices following a snakebite, with a median score of 50% (IQR = 36%–57%). However, only 4.8% (*n* = 33) scored more than 70%. Most respondents said that, after a snakebite, staying calm (66%, *n* = 455), moving slowly away from the snake (75%, *n* = 518) and removing all tight items (e.g., bracelets) on the bitten limb (68%, *n* = 471) would be good practice. However, 69% (*n* = 478) also reported that they would use a tourniquet to prevent the venom from spreading further with the blood circulation, and 71% (*n* = 494) said they would use traditional healing methods to treat snakebite envenoming (Data S5).

Regarding prevention practices, 43.0% (*n* = 297) of the surveyed individuals had fairly adequate knowledge, with a median score of 50% (IQR = 38–69) (Table 3). Over 90% of the participants reported cleaning debris, clearing bushes, filling holes, avoiding marshy and bushy areas, and using a torch at night as effective methods to prevent snakebites. However, a majority of the participants incorrectly deemed the use of snake deterrents, including spraying garlic or onion syrup soup (70%, *n* = 506), snake repellent (53%, *n* = 368), or topical application of herbs (54%, *n* = 375) as effective prevention measures (Data S6).

**TABLE 3:** Knowledge score for snake identification, first aid and prevention of snakebite.

	Type of participant; *n* (%)			
	Community member	Community health worker	Overall	*p*‐value
Number of respondents	379 (54.8)	312 (45.2)	691 (100)	
Knowledge on snake identification^a^				
Median^b^ (IQR)	42 (33–50)	42 (33–50)	42 (33–50)	0.455^c^
Adequate	3 (0.9)	5 (1.7)	8 (1.3)	0.547^d^
Fairly adequate	140 (42.8)	114 (39.6)	254 (41.3)	
Inadequate	184 (56.3)	169 (58.7)	353 (57.4)	
Knowledge on snakebite first aid practices				
Median^c^ (IQR)	50 (43–57)	50 (36–57)	50 (36–57)	0.059^c^
Adequate	17 (4.5)	16 (5.1)	33 (4.8)	
Fairly adequate	201 (53.0)	145 (46.5)	346 (50.1)	0.229^d^
Inadequate	161 (42.5)	151 (48.4)	312 (45.2)	
Knowledge on snakebite prevention practices				
Median^c^ (IQR)	50 (38–69)	50 (38–69)	50 (38–69)	0.525^c^
Adequate	54 (14.2)	45 (14.4)	99 (14.3)	
Fairly adequate	165 (43.5)	132 (42.3)	297 (43.0)	>0.947^d^
Inadequate	160 (42.2)	135 (43.3)	295 (42.7)	
Overall knowledge score				
Median^c^ (IQR)	46 (40–53)	47 (39–54)	46 (39–53)	>0.942^c^
Adequate	2 (0.5)	1 (0.3)	3 (0.4)	
Fairly adequate	141 (37.2)	114 (36.5)	255 (36.9)	>0.949^d^
Inadequate	236 (62.3)	197 (63.2)	433 (62.7)	

^a^ Based on 615 as 76 (52 community members and 24 CHWs) did not consent to see pictures of snakes.

^b^ Median percentage of correct responses (IQR = Interquartile range).

^c^ Mann–Whitney *U* test.

^d^ Fisher's exact test where expected value is 5 or less otherwise Pearson's chi‐squared test.

Overall, the median survey score was 46% (IQR = 39–53), and 62.7% of the respondents (*n* = 433) had inadequate knowledge of snake identification, first aid, and prevention practices. There was no significant difference between community members (62.3%, *n* = 236) and CHWs (63.2%, *n* = 197) (Table 3).

### Predictors of overall knowledge on snake identification, first aid and prevention

Male respondents had a 6.1% points higher overall knowledge score in the adjusted model when compared with female respondents (95% CI: 4.6–7.7; *p* < 0.001). The type of respondent, age, residence during childhood and highest education level were not significantly associated with the overall knowledge score in either the crude or adjusted models (Table 4).

**TABLE 4:** Associations between covariates and overall knowledge score estimated in linear regression analysis.

	Bivariable linear regression		Multivariable linear regression	
Covariate	Estimate (95% CI)	*p*‐value	Estimate (95% CI)	*p*‐value
Respondent type				
Community member	Reference		Reference	
Community health worker	−0.10 (−1.6, 1.4)	>0.900	−0.30 (−1.9, 1.3)	0.710
Sex of respondent				
Female	Reference		Reference	
Male	6.0 (4.5, 7.5)	**<0.001**	6.1 (4.6, 7.7)	**<0.001**
Age of respondent	0.03 (−0.03, 0.08)	0.330	−0.01 (−0.06, 0.05)	0.766
Residence during childhood				
Rural	Reference		Reference	
Urban	0.38 (−3.1, 3.9)	0.834	−0.05 (−3.4, 3.5)	>0.979
Highest education level				
No education/primary	Reference		Reference	
Secondary school/tertiary	−0.07 (−1.6, 1.5)	>0.925	−0.40 (−2.0, 1.2)	0.628

*Note*: Bold indicates significant values.

Abbreviations: CI, confidence interval.

## DISCUSSION

To our knowledge, this is the first study to assess community members' and CHWs' knowledge of snake identification, first‐aid and prevention of snakebites in Malawi. We found that knowledge of snakes was poor, with no difference between community members and CHWs. When pictures of six different snake species were shown, the majority of the surveyed individuals classified all snakes as venomous, despite two being non‐venomous. Similar knowledge gaps have been documented in several other countries where snake identification was also poor [12, 13, 14] and in a previous study that we conducted among facility‐based healthcare workers in the same district [35]. The lack of adequate snake knowledge can have serious implications for health‐seeking behaviour, the administration of effective snakebite treatment and snake conservation.

The negative impact of individuals not being able to identify snakes correctly is compounded by poor knowledge of first aid practices among community members and CHWs, where less than 5% of the participants had adequate knowledge of WHO‐recommended first aid practices following a snakebite, with the majority recognising the use of tourniquets and traditional methods as valid primary first aid measures. Similar findings of first aid knowledge among healthcare workers and the general population have been documented previously in other countries in Asia and Africa [10, 11, 12, 16, 26]. This lack of knowledge could have serious implications, with studies suggesting that improper first‐aid techniques can result in fatalities and long‐term disabilities [3, 4]. While our study did not investigate the use of traditional remedies by CHWs, a previous study on traditional healers found that black stone is not used in Neno district. Instead, traditional healers commonly use herbs and a mixture of a snake's head and herbs as remedies for snakebite management [36]. Knowledge of effective prevention practices was also poor, with less than 15% of respondents having adequate knowledge and over half of the participants considering practices such as hunting and killing snakes or using snake repellents as effective. While knowledge does not directly translate into action, the likelihood of utilising these ineffective and potentially dangerous measures remains high, as shown in Myanmar, Kenya and Bhutan [10, 23, 24]. Improving community members' knowledge of snakes and snakebites is critical if the WHO's goal of decreasing the burden of death and disability resulting from snake envenomation by 50% before 2030 is to be achieved [51, 52].

We found that CHWs lacked formal training on topics related to snakebites, which explains why we did not find significant knowledge differences between CHWs and community members. We advocate for the training of CHWs in first aid and snakebite prevention, which can be done by herpetologists and knowledgeable medical healthcare workers. Trained CHWs could improve the knowledge of community members through the delivery of education sessions, which in turn may improve health‐seeking behaviour, as has been successful in Neno district for other conditions, such as HIV, TB and NCDs [39]. Availability of snake and snakebite‐related materials, for example, in textbooks for school‐going children, posters at public outlets like hospitals, and mass media programs on the radio or other social media platforms, could equally be used to improve the knowledge of both community health workers and other community members. Community engagement campaigns in other settings, including India, Brazil and East Africa, have shown that this approach can be very effective [27, 32, 53]. However, a robust evaluation should be conducted to assess the efficacy of such campaigns in enhancing community member knowledge, specifically around recommended snakebite first aid practices, harmful practices and effective snakebite prevention techniques.

Our study had some limitations. First, we did not evaluate or could not verify whether community members and CHWs used the first‐aid measures described in the event of a snakebite or engaged in similar preventive practices. Nevertheless, the knowledge gaps identified from this study can serve as a valuable foundation for designing an awareness campaign focused on snakebite first aid and prevention. The relationship between first aid knowledge and its translation into practice is an area that has been insufficiently studied and needs further investigation in future research. Second, while the gender distribution among the CHWs (64.1% female) was representative of the underlying distribution, females were slightly overrepresented in our sample of community members (68.6%). As the study was done in November which marks the beginning of the rainy season in Malawi, men were likely to be in the fields preparing land for cropping. In addition, interviews were conducted during working hours between 9:00 AM and 3:00 PM on weekdays which would further explain the higher number of women present in the households included. As knowledge was higher among males in our sample, we may have underestimated the overall knowledge among community members. However, the difference of 6.1% points in the knowledge score is not pronounced. Therefore, a potential underestimation will have been minimal. Future studies should include sampling techniques that ensure fairly representative of both sexes. Third, this study was confined to a small district in southern Malawi. However, we believe the knowledge gaps identified apply to other regions of Malawi due to the lack of country‐wide snakebite education. Finally, we did not match the selection of the community members to that of CHWs (e.g., by age and sex). Therefore, it may be argued that a comparison of these two populations may not be justified. However, we did not see differences in the distribution of demographics between both groups (Table 1) and there were no differences in the knowledge scores, even after adjusting for demographics in the multivariate regression analysis.

### Conclusion

We identified significant knowledge gaps among community members and CHWs regarding local snakes and effective snakebite first aid and prevention practices. The effect of awareness campaigns and other education initiatives could be explored to help improve these gaps. CHWs could play an important role in improving the prevention of snakebites, healthcare‐seeking behaviour and treatment outcomes of patients exposed to this neglected disease in Malawi.

## FUNDING INFORMATION

MBA received funding from the Royal Society of Tropical Medicine and Hygiene (RSTMH) and Wellcome Trust as part of a small research grant initiative (https://rstmh.org/grants/grant-awardees-2021). The funders had no role in study design, data collection and analysis, publication decisions, or manuscript preparation.

## CONFLICT OF INTEREST STATEMENT

The authors declare no conflicts of interest.

## Supporting information


**Data S1.** The STROBE checklist.

**Data S2.** Questionnaire for community health workers and community members' knowledge on identification, first aid and prevention of snakebites.

**Data S3.** Pictures of common venomous and non‐venomous snakes in Southern Malawi.

**Data S4.** Community members and community health workers' knowledge of snake names and their venom status.

**Data S5.** Community members and CHWs' knowledge of snakebite first aid.

**Data S6.** Snakebite prevention knowledge among community members and community health workers.

## Data Availability

The dataset generated and analysed during the current study is available at the Zenodo repository https://zenodo.org/records/13895804.
