# Supplementary material for: Knowledge of local snakes, first‐aid and prevention of snakebites among community health workers and community members in rural Malawi: A cross‐sectional study
Source: Trop Med Int Health. 2024 Dec 17;30(2):84–92. doi: 10.1111/tmi.14071 (PMC11791875; doi:10.1111/tmi.14071)
Supplement: Supplementary file 1 — Data S1. The STROBE checklist. [file TMI-30-84-s005.docx]

**S1: The STROBE checklist**

|  | Item No | Recommendation |
| --- | --- | --- |
| **Title and Abstract** | 1 | (*a*) Indicate the study’s design with a commonly used term in the title or the abstract **– the study design is indicated in the title** |
|  |  | (*b*) Provide in the abstract an informative and balanced summary of what was done and what was found **– the abstract is provided** |
| Introduction | | |
| Background/rationale | 2 | Explain the scientific background and rationale for the investigation being reported **– the introduction section is provided after the abstract** |
| Objectives | 3 | State specific objectives, including any prespecified hypotheses **– the objectives of the study are provided at the end of the introduction** |
| Methods | | |
| Study design | 4 | Present key elements of study design early in the paper **– the study design is presented in the methods section of the paper** |
| Setting | 5 | Describe the setting, locations, and relevant dates, including periods of recruitment, exposure, follow-up, and data collection **- setting, location and relevant dates are provided in the methods** |
| Participants | 6 | (*a*) Give the eligibility criteria, and the sources and methods of selection of participants **– the eligibility is stated in the study population within the methods section** |
| Variables | 7 | Clearly define all outcomes, exposures, predictors, potential confounders, and effect modifiers. Give diagnostic criteria, if applicable **– the data collection and analysis is presented in the methods section** |
| Data sources/ measurement | 8* | For each variable of interest, give sources of data and details of methods of assessment (measurement). Describe comparability of assessment methods if there is more than one group**– the data collection and analysis is presented in the methods section** |
| Bias | 9 | Describe any efforts to address potential sources of bias**– the data collection and analysis is presented in the methods section** |
| Study size | 10 | Explain how the study size was arrived at **- sample size and sampling technique is presented in the methods section** |
| Quantitative variables | 11 | Explain how quantitative variables were handled in the analyses. If applicable, describe which groupings were chosen and why **– data analysis section in the methods section** |
| Statistical methods | 12 | (*a*) Describe all statistical methods, including those used to control for confounding**– data analysis section in the methods section** |
|  |  | (*b*) Describe any methods used to examine subgroups and interactions**– data analysis section in the methods section** |
|  |  | (*c*) Explain how missing data were addressed**– data analysis section in the methods section** |
|  |  | (*d*) If applicable, describe analytical methods taking account of sampling strategy**– data analysis section in the methods section** |
|  |  | (*e*) Describe any sensitivity analyses**– data analysis section in the methods section** |
| Results | | |
| Participants | 13* | (a) Report numbers of individuals at each stage of study—eg numbers potentially eligible, examined for eligibility, confirmed eligible, included in the study, completing follow-up, and analysed **– socio demographic characteristic section is presented in the results** |
|  |  | (b) Give reasons for non-participation at each stage |
|  |  | (c) Consider use of a flow diagram |
| Descriptive data | 14* | (a) Give characteristics of study participants (eg demographic, clinical, social) and information on exposures and potential confounders**– socio demographic characteristic section is presented in the results** |
|  |  | (b) Indicate number of participants with missing data for each variable of interest |
| Outcome data | 15* | Report numbers of outcome events or summary measures **– sections on knowledge of snake identification, first aid and prevention are presented in the results section** |
| Main results | 16 | (*a*) Give unadjusted estimates and, if applicable, confounder-adjusted estimates and their precision (eg, 95% confidence interval). Make clear which confounders were adjusted for and why they were included **– This has been presented in the regression models within the results section** |
|  |  | (*b*) Report category boundaries when continuous variables were categorized **– Categories of knowledge as adequate, firstly adequate and inadequate are presented in the results section** |
|  |  | (*c*) If relevant, consider translating estimates of relative risk into absolute risk for a meaningful time period |
| Other analyses | 17 | Report other analyses done—eg analyses of subgroups and interactions, and sensitivity analyses **Not applicable** |
| Discussion | | |
| Key results | 18 | Summarise key results with reference to study objectives **– this is presented in the first paragraph of the discussion section** |
| Limitations | 19 | Discuss limitations of the study, taking into account sources of potential bias or imprecision. Discuss both direction and magnitude of any potential bias **– A paragraph on limitation is presented in the discussion section.** |
| Interpretation | 20 | Give a cautious overall interpretation of results considering objectives, limitations, multiplicity of analyses, results from similar studies, and other relevant evidence **– this is thoroughly discussed** |
| Generalisability | 21 | Discuss the generalisability (external validity) of the study results **-this has be presented in the discussion section** |
| Other information | | |
| Funding | 22 | Give the source of funding and the role of the funders for the present study and, if applicable, for the original study on which the present article is based **– the funding statement is presented** |
