# Supplementary material for: Knowledge of local snakes, first‐aid and prevention of snakebites among community health workers and community members in rural Malawi: A cross‐sectional study
Source: Trop Med Int Health. 2024 Dec 17;30(2):84–92. doi: 10.1111/tmi.14071 (PMC11791875; doi:10.1111/tmi.14071)
Supplement: Supplementary file 2 — Data S2. Questionnaire for community health workers and community members' knowledge on identification, first aid and prevention of snakebites. [file TMI-30-84-s003.docx]

**S2: Questionnaire for community health workers and community members' knowledge on identification, first aid and prevention of snakebites**

| **Type:** Community member  Community health worker | | | | |
| --- | --- | --- | --- | --- |
| **Sn** | **Question** | **Options** | | **Skip** |
| **A** | **Socio-demographic Characteristics** | | |  |
| A1. | Sex | Male----------------------------------------1  Female-------------------------------------2 | |  |
| A2. | Age in years | Years \|___\|___\|  Don’t Know------------------------------99 | |  |
| A3 | Village name |  | |  |
| A4 | Traditional Authority | Dambe----------------------------------------1  Chekucheku----------------------------------2  Mlauli-----------------------------------------3  Symon----------------------------------------4 | |  |
| A5 | Religion | Christianity ----------------------------------1  Muslim-----------------------------------------2  Other--------------------------------------------3 | |  |
| A6 | Where did you spend most of your childhood | Rural -------------------------------------------1  Urban-------------------------------------------2 | |  |
| A7. | What is your level of education | No formal education -------------------------1  Primary School -------------------------------2  Secondary School-----------------------------3  Tertiary-----------------------------------------4 | |  |
| A8 | The respondent marital status | Married / living together --------------------1  Divorced / separated -------------------------2  Widowed --------------------------------------3  Single-------------------------------------------4 | |  |
| A9 | Total number of females in the household |  | |  |
| A10 | Total number of males in the household |  | |  |
| A11 | Time to nearest health facility | <30 minutes ---------------------------------1  Between 30 and 1 hour---------------------2  >1 hour---------------------------------------3  Don’t know----------------------------------4 | |  |
| **B** | **Snake identification** | | | |
| B1 | Are you comfortable seeing pictures of snakes? | Yes ----------------------------------------1  No------------------------------------------2 | | If no skip to C1 |
| B2 | What is the name of the snake labelled A? (Puff adder) | Don’t know----------------------------99 | |  |
| B3 | Is the snake venomous? | Yes ----------------------------------------1  No-----------------------------------------2  Don’t know------------------------------3 | |  |
| B4 | What is the name of the snake labelled B (Black Mamba) | Don’t know----------------------------99 | |  |
| B5 | Is the snake venomous | Yes ----------------------------------------1  No-----------------------------------------2  Don’t know------------------------------3 | |  |
| B6 | What is the name of the snake labelled C? (Common House snake) | Don’t know----------------------------99 | |  |
| B7 | Is the snake venomous? | Yes ----------------------------------------1  No-----------------------------------------2  Don’t know------------------------------3 | |  |
| B8 | What is the name of the snake labelled D (Oates’ Vine twig) | Don’t know----------------------------99 | |  |
| B9 | Is the snake venomous | Yes ----------------------------------------1  No-----------------------------------------2  Don’t know------------------------------3 | |  |
| B10 | What is the name of the snake labelled E? (Spotted Bush snake) | Don’t know----------------------------99 | |  |
| B11 | Is the snake venomous? | Yes ----------------------------------------1  No-----------------------------------------2  Don’t know------------------------------3 | |  |
| B12 | What is the name of the snake labelled F (Mozambique spitting Cobra) | Don’t know----------------------------99 | |  |
| B13 | Is the snake venomous | Yes ----------------------------------------1  No-----------------------------------------2  Don’t know------------------------------3 | |  |
| **C** | **Would the following act as a first-aid procedure during snakebite?**  **Note:**  ******* *Proper first aid practices WHO Guidelines for the Prevention and Clinical Management*  ****** *Harmful first-aid practices* | | |  |
| C1 | Stay calm and reassure the bitten person* | Yes-----------------------------------------------1  No------------------------------------------------2  Don’t Know------------------------------------3 | |  |
| C2 | Move slowly away from the snake* | Yes-----------------------------------------------1  No------------------------------------------------2  Don’t Know------------------------------------3 | |  |
| C3 | Attack or kill the snake** | Yes-----------------------------------------------1  No------------------------------------------------2  Don’t Know------------------------------------3 | |  |
| C4 | Rub the eyes in case of spiting snakes** | Yes-----------------------------------------------1  No------------------------------------------------2  Don’t Know------------------------------------3 | |  |
| C5 | Rise venom in the eyes with running water in case of spitting snakes* | Yes-----------------------------------------------1  No------------------------------------------------2  Don’t Know------------------------------------3 | |  |
| C6 | Leave the wound are (or bite mark) alone* | Yes-----------------------------------------------1  No------------------------------------------------2  Don’t Know------------------------------------3 | |  |
| C7 | Wash, cut or suck the wound** | Yes-----------------------------------------------1  No------------------------------------------------2  Don’t Know------------------------------------3 | |  |
| C8 | Tie the affected area to stop blood circulation** | Yes-----------------------------------------------1  No------------------------------------------------2  Don’t Know------------------------------------3 | |  |
| C9 | Remove all the tight items around the affected area* | Yes-----------------------------------------------1  No------------------------------------------------2  Don’t Know------------------------------------3 | |  |
| C10 | Lay the patient on his / her back** | Yes-----------------------------------------------1  No------------------------------------------------2  Don’t Know------------------------------------3 | |  |
| C11 | Lay the patient on his / her side and reduce the movement of the affected area* | Yes-----------------------------------------------1  No------------------------------------------------2  Don’t Know------------------------------------3 | |  |
| C12 | Rush to the nearest health facility for medical treatment* | Yes-----------------------------------------------1  No------------------------------------------------2  Don’t Know------------------------------------3 | |  |
| C13 | Panic** | Yes-----------------------------------------------1  No------------------------------------------------2  Don’t Know------------------------------------3 | |  |
| C14 | Use traditional methods or any unsafe treatment** | Yes-----------------------------------------------1  No------------------------------------------------2  Don’t Know------------------------------------3 | |  |
| **D** | **Would the following methods help to prevent snakebites?**  **Note:**  ******* *Proven good practices according to WHO Guidelines for the Prevention and Clinical Management*  ****** *Unproven prevention practices* | | | |
| D1 | Spraying phenol** | Yes-----------------------------------------------1  No------------------------------------------------2  Don’t Know------------------------------------3 |  | |
| D2 | Hunting and killing snakes** | Yes-----------------------------------------------1  No------------------------------------------------2  Don’t Know------------------------------------3 |  | |
| D3 | Cleaning and clearing bushes, and debris laying on the ground* | Yes-----------------------------------------------1  No------------------------------------------------2  Don’t Know------------------------------------3 |  | |
| D4 | Spraying kerosene ** | Yes-----------------------------------------------1  No------------------------------------------------2  Don’t Know------------------------------------3 |  | |
| D5 | Use of protective equipment* | Yes-----------------------------------------------1  No------------------------------------------------2  Don’t Know------------------------------------3 |  | |
| D6 | Cover up holes in surrounding* | Yes-----------------------------------------------1  No------------------------------------------------2  Don’t Know------------------------------------3 |  | |
| D7 | Avoid marshy and bushy area* | Yes-----------------------------------------------1  No------------------------------------------------2  Don’t Know------------------------------------3 |  | |
| D8 | Spraying Alcohol** | Yes-----------------------------------------------1  No------------------------------------------------2  Don’t Know------------------------------------3 |  | |
| D9 | Spraying garlic or onion syrup/soup** | Yes-----------------------------------------------1  No------------------------------------------------2  Don’t Know------------------------------------3 |  | |
| D10 | Praying to Gods** | Yes-----------------------------------------------1  No------------------------------------------------2  Don’t Know------------------------------------3 |  | |
| D11 | Spraying snake repellent* | Yes-----------------------------------------------1  No------------------------------------------------2  Don’t Know------------------------------------3 |  | |
| D12 | Planting snake repellent at home** | Yes-----------------------------------------------1  No------------------------------------------------2  Don’t Know------------------------------------3 |  | |
| D13 | Sleep off the ground and use mosquito nets to avoid snakebites during sleep* | Yes-----------------------------------------------1  No------------------------------------------------2  Don’t Know------------------------------------3 |  | |
| D14 | Use a torch outside at night* | Yes-----------------------------------------------1  No------------------------------------------------2  Don’t Know------------------------------------3 |  | |
| D15 | Using snake repellant ointments** | Yes--------------------------------------------1  No--------------------------------------------2  I don’t know--------------------------------3 |  | |
| D16 | I use herbals on me that prevent snakes from biting me** | Yes--------------------------------------------1  No--------------------------------------------2  I don’t know--------------------------------3 |  | |
| **D** | **Training of the snakebite – only for Community Health Workers** | |  | |
| D1 | Number of years of practice as CHW (to the nearest whole number) | ______________________________ |  | |
| D2 | Reporting Health facility | Hospital --------------------------------------1  Health Centre--------------------------------2 |  | |
| D3 | In the past year, have you offered any education on snakes and snakebite management and prevention in the household you visit | Yes-----------------------------------------------1  No -----------------------------------------------2  Can’t remember-------------------------------3 |  | |
| D4 | For yes; how many times | Once--------------------------------------------1  Twice-------------------------------------------2  Thrice-------------------------------------------3  4 – 9 times ------------------------------------4  > 10 times-------------------------------------5 |  | |
| D5 | Have you received any snakebite first aid training | Yes--------------------------------------------1  No--------------------------------------------2 |  | |
| D6 | When was the training received | <6 months---------------------------------1  >=6 months and <2 2years--------------2  >=2 years----------------------------------3 |  | |
