# Supplementary material for: Knowledge of local snakes, first‐aid and prevention of snakebites among community health workers and community members in rural Malawi: A cross‐sectional study
Source: Trop Med Int Health. 2024 Dec 17;30(2):84–92. doi: 10.1111/tmi.14071 (PMC11791875; doi:10.1111/tmi.14071)
Supplement: Supplementary file 3 — Data S3. Pictures of common venomous and non‐venomous snakes in Southern Malawi. [file TMI-30-84-s004.docx]

**S3 Pictures of Common Venomous and Non-Venomous Snakes in Southern Malawi**

| English name: Puff Adder (venomous)  Scientific name: *Bitis arietans*  Chichewa names: Mphiri, Mpiri, Chiphiri  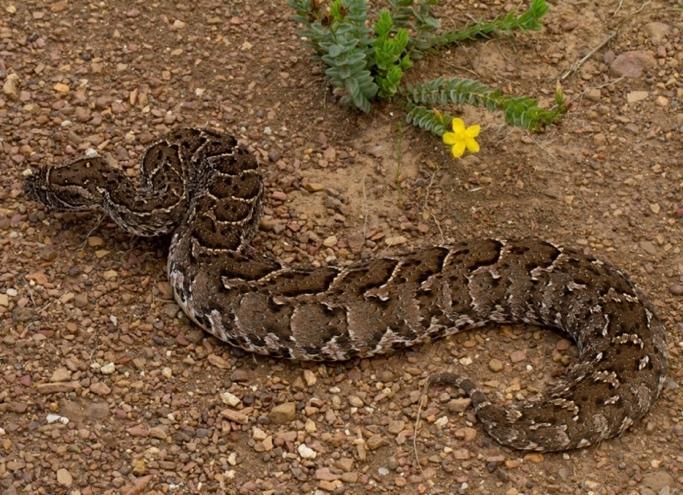 | English name: Black Mamba (venomous)  Scientific name: *Dendroaspis polylepis*  Chichewa name called mamba  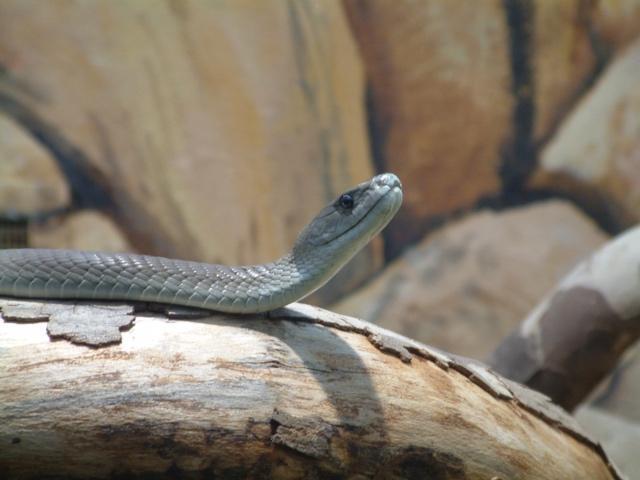 |
| --- | --- |
| English name: Common House Snake (non-venomous)  Scientific name: *Boaedon capensis*  Chichewa name: Chankusa, Chakusa.  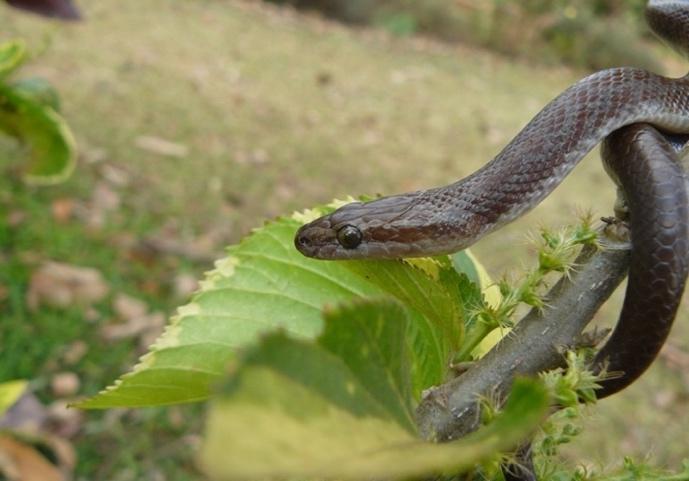 | English name: Oates’ vine twig (venomous)  Scientific name: *Thelotornis oastesi;*  Chichewa name: Nakalikukuti, Kalikukuti or Kamutimuti.  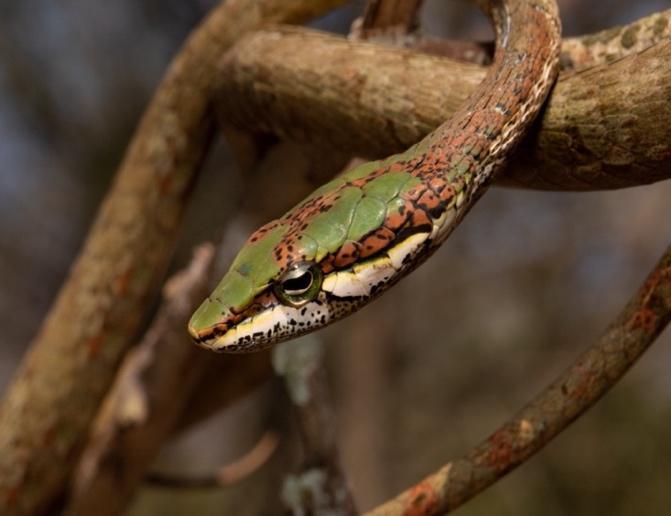 |
| English name: Spotted bush snake (non-venomous)  Scientific name: *Philothamnus semivariegatus*  Chichewa name: Camasamba, Cipota Masamba, Njokansipu and Namasamba.  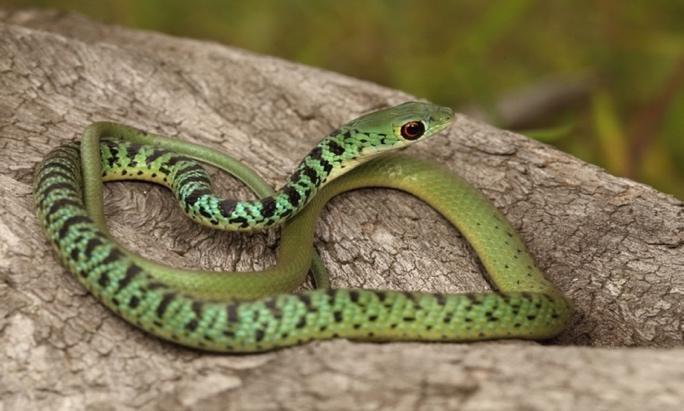 | English name: Mozambique Spitting Cobra (venomous)  Scientific name: Naja mossambica  Chichewa name: Mamba and Citawo.  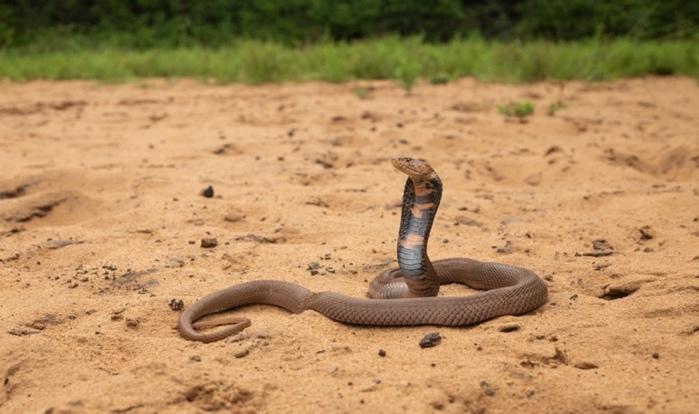 |
