# Supplementary material for: Knowledge of local snakes, first‐aid and prevention of snakebites among community health workers and community members in rural Malawi: A cross‐sectional study
Source: Trop Med Int Health. 2024 Dec 17;30(2):84–92. doi: 10.1111/tmi.14071 (PMC11791875; doi:10.1111/tmi.14071)
Supplement: Supplementary file 4 — Data S4. Community members and community health workers' knowledge of snake names and their venom status. [file TMI-30-84-s002.docx]

**S4 Community members and community health workers’ knowledge of snake names and their venom status**

|  | Type of respondent;  n (%) | | | |
| --- | --- | --- | --- | --- |
|  | **Community member** | **Community health worker** | **Overall** | |
| Number of respondents^1^ | 327 (86) | 288 (92) | 615 (89) | |
| **Venomous snakes** | | | | |
| Puff adder | | | | |
| *Correct* | 285 (87) | 267 (93) | 552 (90) | |
| *Incorrect* | 32 (9.8) | 17 (5.9) | 49 (8.0) | |
| *Don’t know* | 10 (3.1) | 4 (1.4) | 14 (2.3) | |
| Is the snake venomous? | |  |  | |
| *Venomous* | 312 (95) | 280 (97) | 592 (96) | |
| *Non-venomous* | 1 (0.3) | 1 (0.3) | 2 (0.3) | |
| *Don’t know* | 14 (4.3) | 7 (2.4) | 21 (3.4) | |
| Black mamba | | | | |
| *Correct* | 91 (28) | 49 (17) | 140 (23) | |
| *Incorrect* | 176 (54) | 170 (59) | 346 (56) | |
| *Don’t know* | 60 (18) | 69 (24) | 129 (21) | |
| Is the snake venomous? | | | | |
| *Venomous* | 282 (86) | 231 (80) | 513 (83) |  |
| *Non-venomous* | 5 (1.5) | 6 (2.1) | 11 (1.8) |  |
| *Don’t know* | 40 (12) | 51 (18) | 91 (15) |  |
| Snake Oates’ vine twig | | | | |
| *Correct* | 75 (23) | 53 (18) | 128 (21) | |
| *Incorrect* | 97 (30) | 93 (32) | 190 (31) | |
| *Don’t know* | 155 (47) | 142 (49) | 297 (48) | |
| Is the snake venomous? |  |  |  | |
| *Venomous* | 178 (54) | 152 (53) | 330 (54) | |
| *Non-venomous* | 17 (5.2) | 13 (4.5) | 30 (4.9) | |
| *Don’t know* | 132 (40) | 123 (43) | 255 (41) | |
| Mozambique spitting cobra | | | | |
| *Correct* | 151 (46) | 158 (55) | 309 (50) | |
| *Incorrect* | 83 (25) | 72 (25) | 155 (25) | |
| *Don’t know* | 93 (28) | 58 (20) | 151 (25) | |
| Is the snake venomous? |  |  |  | |
| *Venomous* | 261 (80) | 245 (85) | 506 (82) | |
| *Non-venomous* | 4 (1.2) | 1 (0.3) | 5 (0.8) | |
| *Don’t know* | 62 (19) | 42 (15) | 104 (17) | |
| **Non-venomous snakes** | | | | |
| Common house snake |  |  |  | |
| *Correct* | 7 (2.1) | 3 (1.0) | 10 (1.6) | |
| *Incorrect* | 192 (59) | 161 (56) | 353 (57) | |
| *Don’t know* | 128 (39) | 124 (43) | 252 (41) | |
| Is the snake venomous? | | | | |
| *Venomous* | 219 (67) | 189 (66) | 408 (66) | |
| *Non-venomous* | 11 (3.4) | 8 (2.8) | 19 (3.1) | |
| *Don’t know* | 97 (30) | 91 (32) | 188 (31) | |
| Spotted bush snake | |  |  | |
| *Correct* | 18 (5.5) | 16 (5.6) | 34 (5.5) | |
| *Incorrect* | 222 (68) | 197 (68) | 419 (68) | |
| *Don’t know* | 87 (27) | 75 (26) | 162 (26) | |
| Is the snake venomous? | | | | |
| *Venomous* | 261 (80) | 234 (81) | 495 (80) | |
| *Non-venomous* | 11 (3.4) | 1 (0.3) | 12 (2.0) | |
| *Don’t know* | 55 (17) | 53 (18) | 108 (18) | |
| ^1^ Percentage calculated as the total number of community members, community health workers, and total surveyed population who answered questions on snake identification divided by the total number of individuals in those subgroups included in the cross-sectional survey. | | | | |
