# Supplementary material for: Knowledge of local snakes, first‐aid and prevention of snakebites among community health workers and community members in rural Malawi: A cross‐sectional study
Source: Trop Med Int Health. 2024 Dec 17;30(2):84–92. doi: 10.1111/tmi.14071 (PMC11791875; doi:10.1111/tmi.14071)
Supplement: Supplementary file 5 — Data S5. Community members and CHWs' knowledge of snakebite first aid. [file TMI-30-84-s001.docx]

**S5 Community members and CHWs' knowledge of snakebite first aid**

|  | Type of respondent;  n (%) | | |
| --- | --- | --- | --- |
|  | **Community member** | **Community health worker** | **Overall** |
| Number of respondents | 379 (55) | 312 (45) | 691 (100) |
| **Good practices^1^** | | | |
| Stay calm and reassure the bitten person | | |  |
| *Yes* | 247 (65) | 208(67) | 455(66) |
| *No* | 105 (28) | 81(26) | 186(27) |
| *Don’t know* | 27 (7.1) | 23(7.4) | 50(7.2) |
| Move slowly away from the snake | | |  |
| *Yes* | 283 (75) | 235(75) | 518(75) |
| *No* | 80 (21) | 67(21) | 147(21) |
| *Don’t know* | 16 (4.2) | 10(3.2) | 26(3.8) |
| Rinse venom in the eyes with running water in case of spitting snakes | | | |
| *Yes* | 133 (35) | 99(32) | 232(34) |
| *No* | 188 (50) | 130(42) | 318(46) |
| *Don’t know* | 58 (15) | 83(27) | 141(20) |
| Leave the wound or bite mark alone | | | |
| *Yes* | 157 (41) | 127(41) | 284(41) |
| *No* | 174 (46) | 106(34) | 280(41) |
| *Don’t know* | 48 (13) | 79(25) | 127(18) |
| Remove all the tight items around the affected area | | | |
| *Yes* | 272 (72) | 199 (64) | 471 (68) |
| *No* | 66 (17) | 44 (14) | 110 (16) |
| *Don’t know* | 41 (11) | 69 (22) | 110 (16) |
| Lay the patient on his or her side and reduce the movement of the affected area | | | |
| *Yes* | 159 (42) | 94 (30) | 253 (37) |
| *No* | 73 (19) | 58 (19) | 131 (19) |
| *Don’t know* | 147 (39) | 160 (51) | 307 (44) |
| Rush to the nearest health facility for medical treatment | | | |
| *Yes* | 358 (94) | 297 (95) | 655 (95) |
| *No* | 16 (4.2) | 10 (3.2) | 26 (3.8) |
| *Don’t know* | 5 (1.3) | 5 (1.6) | 10 (1.4) |
| **Bad practices^2^** | | | |
| Attack or kill the snake | | |  |
| *Yes* | 345 (91) | 288 (92) | 633 (92) |
| *No* | 26 (6.9) | 21 (6.7) | 47 (6.8) |
| *Don’t know* | 8 (2.1) | 3 (1.0) | 11 (1.6) |
| Rub the eyes in case of spiting snakes | | |  |
| *Yes* | 67 (18) | 41 (13) | 108 (16) |
| *No* | 252 (66) | 210 (67) | 462 (67) |
| *Don’t know* | 60 (16) | 61 (20) | 121 (18) |
| Wash, cut or suck the wound | | |  |
| *Yes* | 134 (35) | 105 (34) | 239 (35) |
| *No* | 218 (58) | 163 (52) | 381 (55) |
| *Don’t know* | 27 (7.1) | 44 (14) | 71 (10) |
| Tie the affected area to stop blood circulation (tourniquet) | | |  |
| *Yes* | 252 (66) | 226 (72) | 478 (69) |
| *No* | 103 (27) | 68 (22) | 171 (25) |
| *Don’t know* | 24 (6.3) | 18 (5.8) | 42 (6.1) |
| Lay the patient on his or her back | | |  |
| *Yes* | 108 (28) | 61 (20) | 169 (24) |
| *No* | 101 (27) | 78 (25) | 179 (26) |
| *Don’t know* | 170 (45) | 173 (55) | 343 (50) |
| Panic as patient will die anyway | | |  |
| *Yes* | 203 (54) | 155 (50) | 358 (52) |
| *No* | 168 (44) | 151 (48) | 319 (46) |
| *Don’t know* | 8 (2.1) | 6 (1.9) | 14 (2.0) |
| Use traditional methods | | |  |
| *Yes* | 281(74) | 213 (68) | 494 (71) |
| *No* | 90 (24) | 84 (27) | 174 (25) |
| *Don’t know* | 8 (2.1) | 15 (4.8) | 23 (3.3) |
| ^1^ Good practices according to Health Action International snakebite first aid leaflet.  ^2^ Bad practices according to Health Action International snakebite first aid leaflet. | | | |
