# Supplementary material for: Knowledge of local snakes, first‐aid and prevention of snakebites among community health workers and community members in rural Malawi: A cross‐sectional study
Source: Trop Med Int Health. 2024 Dec 17;30(2):84–92. doi: 10.1111/tmi.14071 (PMC11791875; doi:10.1111/tmi.14071)
Supplement: Supplementary file 6 — Data S6. Snakebite prevention knowledge among community members and community health workers. [file TMI-30-84-s006.docx]

**S6: Snakebite prevention knowledge among community members and community health workers**

|  | Type of respondent;  n (%) | | |
| --- | --- | --- | --- |
|  | **Community member** | **Community health worker** | **Overall** |
| Number of respondents | 379 (55) | 312 (45) | 691 (100) |
| **Recommended practices^1^** | | | |
| Clearing bushes and debris laying on the ground | | | |
| *Yes* | 376 (99) | 310 (99) | 686 (99) |
| *No* | 3 (0.8) | 2 (0.6) | 5 (0.7) |
| *Don’t know* | 0 (0) | 0 (0) | 0 (0) |
| Use of protective equipment | | | |
| *Yes* | 258 (68) | 178 (57) | 436 (63) |
| *No* | 56 (15) | 55 (18) | 111 (16) |
| *Don’t know* | 65 (17) | 79 (25) | 144 (21) |
| Cover up holes in surrounding | | | |
| *Yes* | 365 (96) | 303 (97) | 668 (97) |
| *No* | 11 (2.9) | 8 (2.6) | 19 (2.7) |
| *Don’t know* | 3 (0.8) | 1 (0.3) | 4 (0.6) |
| Avoid marshy and bushy areas | | | |
| *Yes* | 350 (92) | 298 (96) | 648 (94) |
| *No* | 19 (5.0) | 12 (3.8) | 31 (4.5) |
| *Don’t know* | 10 (2.6) | 2 (0.6) | 12 (1.7) |
| Sleep off the ground and use mosquito nets to avoid snakebites during sleep | | | |
| *Yes* | 200 (53) | 117 (38) | 317 (46) |
| *No* | 144 (38) | 124 (40) | 268 (39) |
| *Don’t know* | 35 (9.2) | 71 (23) | 106 (15) |
| Use a torch outside at night | | | |
| *Yes* | 367 (97) | 306 (98) | 673 (97) |
| *No* | 11 (2.9) | 6 (1.9) | 17 (2.5) |
| *Don’t know* | 1 (0.3) | 0 (0) | 1 (0.1) |
| **Unproven practices** | | | |
| Spraying phenol |  |  |  |
| *Yes* | 125 (33) | 73 (23) | 198 (29) |
| *No* | 147 (39) | 137 (44) | 284 (41) |
| *Don’t know* | 107 (28) | 102 (33) | 209 (30) |
| Hunting and killing snakes | | | |
| *Yes* | 146 (39) | 125 (40) | 271 (39) |
| *No* | 216 (57) | 181 (58) | 397 (57) |
| *Don’t know* | 17 (4.5) | 6 (1.9) | 23 (3.3) |
| Spraying kerosene | | | |
| *Yes* | 76 (20) | 37 (12) | 113 (16) |
| *No* | 184 (49) | 159 (51) | 343 (50) |
| *Don’t know* | 119 (31) | 116 (37) | 235 (34) |
| Spraying Alcohol |  |  |  |
| *Yes* | 13 (3.4) | 8 (2.6) | 21 (3.0) |
| *No* | 223 (59) | 171 (55) | 394 (57) |
| *Don’t know* | 143 (38) | 133 (43) | 276 (40) |
| Spraying garlic or onion syrup soup | | | |
| *Yes* | 282 (74) | 224 (72) | 506 (73) |
| *No* | 66 (17) | 54 (17) | 120 (17) |
| *Don’t know* | 31 (8.2) | 34 (11) | 65 (9.4) |
| Praying to God |  |  |  |
| *Yes* | 308 (81) | 248 (79) | 556 (80) |
| *No* | 60 (16) | 57 (18) | 117 (17) |
| *Don’t know* | 11 (2.9) | 7 (2.2) | 18 (2.6) |
| Planting plants thought to repel snakes at home | | | |
| *Yes* | 264 (70) | 217 (70) | 481 (70) |
| *No* | 62 (16) | 43 (14) | 105 (15) |
| *Don’t know* | 53 (14) | 52 (17) | 105 (15) |
| Using snake repellent ointments | | | |
| *Yes* | 115 (30) | 57 (18) | 172 (25) |
| *No* | 125 (33) | 112 (36) | 237 (34) |
| *Don’t know* | 139 (37) | 143 (46) | 282 (41) |
| Spraying snake repellent | | | |
| *Yes* | 219 (58) | 149 (48) | 368 (53) |
| *No* | 94 (25) | 81 (26) | 175 (25) |
| *Don’t know* | 66 (17) | 82 (26) | 148 (21) |
| Topical application of herbs to prevent snake bites | | | |
| *Yes* | 232 (61) | 143 (46) | 375 (54) |
| *No* | 111 (29) | 130 (42) | 241 (35) |
| *Don’t know* | 36 (9.5) | 39 (12) | 75 (11) |
| ^1^ Recommended good practice to reduce snakebite according to WHO Guidelines for the Prevention and Clinical Management of snakebite in Africa and other studies | | | |
